# Case Report: Difficulties in the Treatment of a 12-Year-Old Patient With Homozygous Familial Hypercholesterolemia, Compound Heterozygous Form − 5 Years Follow-Up

**DOI:** 10.3389/fcvm.2021.743341

**Published:** 2021-10-08

**Authors:** Lyudmila Vladimirova-Kitova, Spas Kitov, Mihail Ganev, Lubov Chochkova-Bukova

**Affiliations:** ^1^First Department of Internal Diseases, Section of Cardiology, Medical University of Plovdiv, Plovdiv, Bulgaria; ^2^Clinic of Cardiology, St. George University Hospital, Plovdiv, Bulgaria; ^3^Department of Medical Genetics, Medical University of Sofia, Sofia, Bulgaria; ^4^Department of Paediatrics and Medical Genetics, Medical Faculty, Medical University of Plovdiv, Plovdiv, Bulgaria

**Keywords:** case report, homozygous familial hypercholesterolemia, child, Evolocumab, COVID-19

## Abstract

The literature review we conducted reveals the limited use of proprotein convertase subtilisin/kexin type 9-inhibitors (PCSK9i) in children with familial hypercholesterolemia (FH). In 2015, a 10-year-old boy presented with round, xanthochromic lesions on his right knee and elbow. The values of total and LDL-cholesterol (LDL-C)−18 and 15 mmol/l, respectively—along with normal triglycerides and HDL-cholesterol (HDL-C) confirmed the lesions were xanthomas. The data suggested a homozygous form of FH. The level of lipoprotein (a) was high: 270 mg/dl. Initial treatment, based on European recommendations, included Atorvastatin 20 mg and Ezetimibe 10 mg and led to a decrease in LDL-C by 46% for 5 months; however, the patient developed severe statin intolerance. Atorvastatin was replaced with Rosuvastatin 10 mg, but the symptoms persisted. Success was achieved by switching to an intermittent regimen: Rosuvastatin 10 mg three times a week with a daily intake of Ezetimibe 10 mg. However, the results were far from the desired LDL target. LDL-apheresis was advisable, but unfortunately, it is not performed in Bulgaria. In May 2017, a genetic analysis [two pathological mutations within the *LDLR* gene: c.1519A>G; p.(Lys507Glu) and c.2403_2406del; p.(Leu802Alafs^*^126)] confirmed the initial diagnosis: the patient had homozygous FH with compound heterozygosity indeed. Having turned 12 in September 2017, the patient was eligible for treatment with a PCSK9i: Evolocumab 140 mg. The mean reduction of LDL-C with the triple combination reached a reduction of 52.17% for the whole 2-year period. The LDL target was reached in January 2020. The triple therapy significantly reduced Apolipoprotein B by 29.16%. No statistically significant difference was found in Lp (a) levels (*p* > 0.05) Our clinical case demonstrates that the triple lipid-lowering combination in a patient with compound heterozygous FH is a good therapeutic option for reaching the LDL-target.

## Introduction

The homozygous forms of familial hypercholesterolemia (HoFH) are rare (incidence is often cited to be 1:1,000,000 although estimations show that it could be as frequent as 1 in 160,000–320,000 people) and pose a significant therapeutic challenge (1, 2). In both HoFH forms (true homozygous and compound heterozygous) low-density lipoprotein (LDL) target levels are difficult to reach without treatment with LDL-apheresis, Mipomersen, or Lomitapide. The literature review reveals a limited use of proprotein convertase subtilisin/kexin type 9 inhibitors (PCSK9i) in children with FH (1–5). According to the European recommendations, diagnostic criteria of HoFH include untreated LDL-cholesterol (LDL-C) levels >13 mmol/L (500 mg/dL) and pediatric manifestations such as premature coronary heart disease, aortic valve disease, and tendon xanthomas in the hands and Achilles tendons (4, 5). The American Heart Association guidelines give a simplified clinical classification of HoFH: (1) appearance of xanthomas before the age of 10; (2) LDL-C >13 mmol/l before treatment or >7.76 mmol/l despite treatment; (3) phenotype features or documented hypercholesterolemia in both parents (6).

## Case Report

### Case Description

In mid-2015, a 9-year-old boy first sought medical attention due to the occurrence of round, xanthochromic lesions on his right knee and right elbow as shown in the following pictures. Progression of the lesions was gradual and non-traumatic.

**Figure 1:**
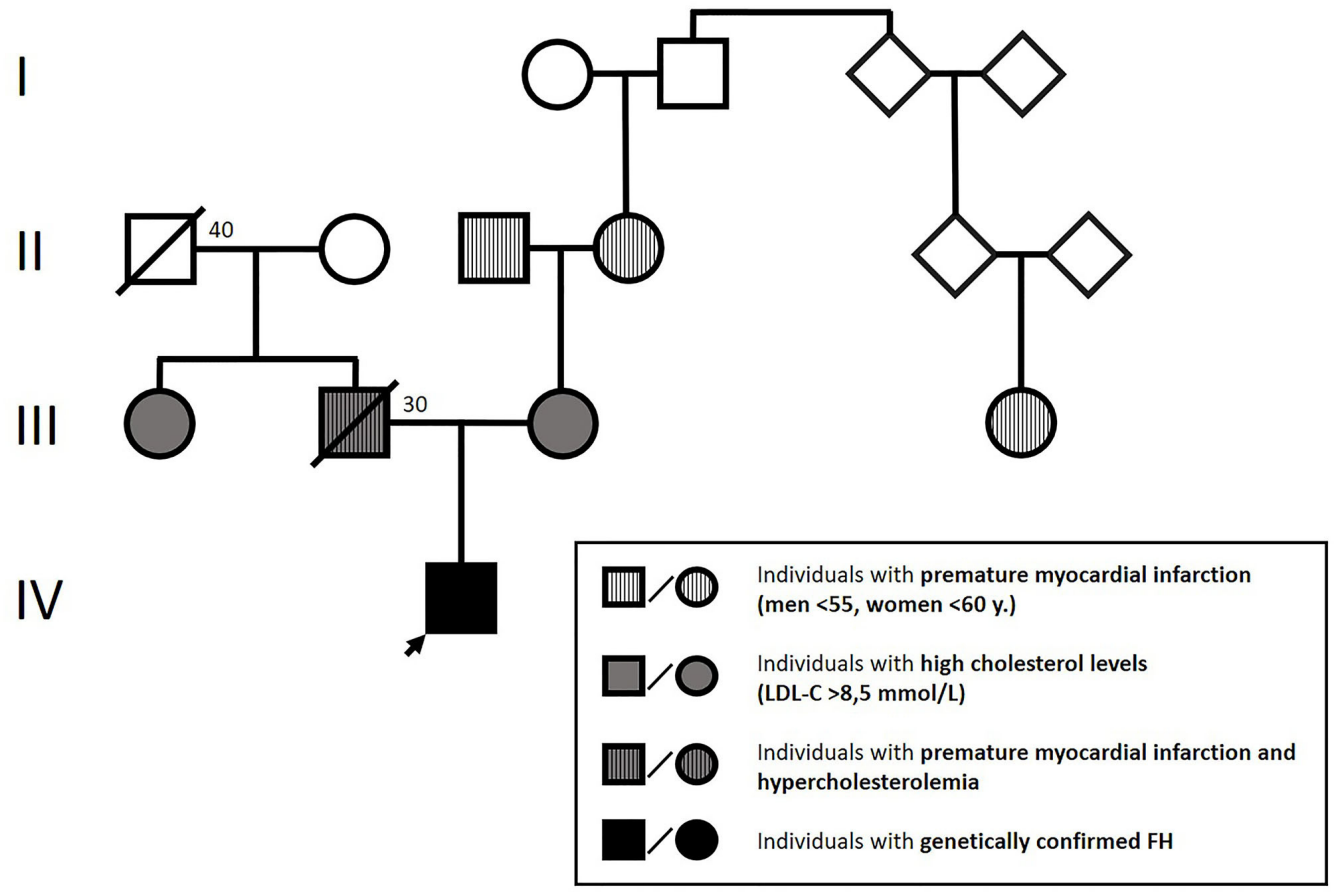
Patient's family tree according to the boy's mother.

Physical examination showed height: 145 cm, weight: 46 kg, body mass index (BMI): 18.62 kg/m^2^. The patient presented with blood pressure of 115/70 mmHg and heart rate of 95 bpm. No pathologic heart sounds were heard on auscultation; peripheral pulsations were within normal limits. Blood lipid analysis revealed extremely elevated total and LDL-C levels (18 and 15 mmol/l, respectively) with normal triglycerides (1.3 mmol/l) and HDL-C (1.4 mmol/l). Further laboratory tests revealed an Apolipoprotein-B/Apolipoprotein-A_1_ ratio of 2.9 (Apolipoprotein-B: 3.5 g/l, Apolipoprotein-A_1_: 1.4 g/l), which confirmed that the abovementioned lesions were xanthomas. The patient had normal thyroid function, normal vitamin D_3_ levels, and no jaundice or anemia. We also found high levels of lipoprotein (a) (Lp(a): 270 mg/dl), which was measured by an immunoturbidimetric assay. Family history: The available data on the affected family members are presented in Figure 1.

According to the Simon Broome criteria, the boy was classified as having a definite FH (4, 5). Based on the early age of occurrence of xanthomas, extremely elevated LDL cholesterol levels, the presence of hypercholesterolemia in both parents, and family history of premature ischemic heart disease, we concluded that the condition was in its homozygous form (4–6).

**Figure 2:**
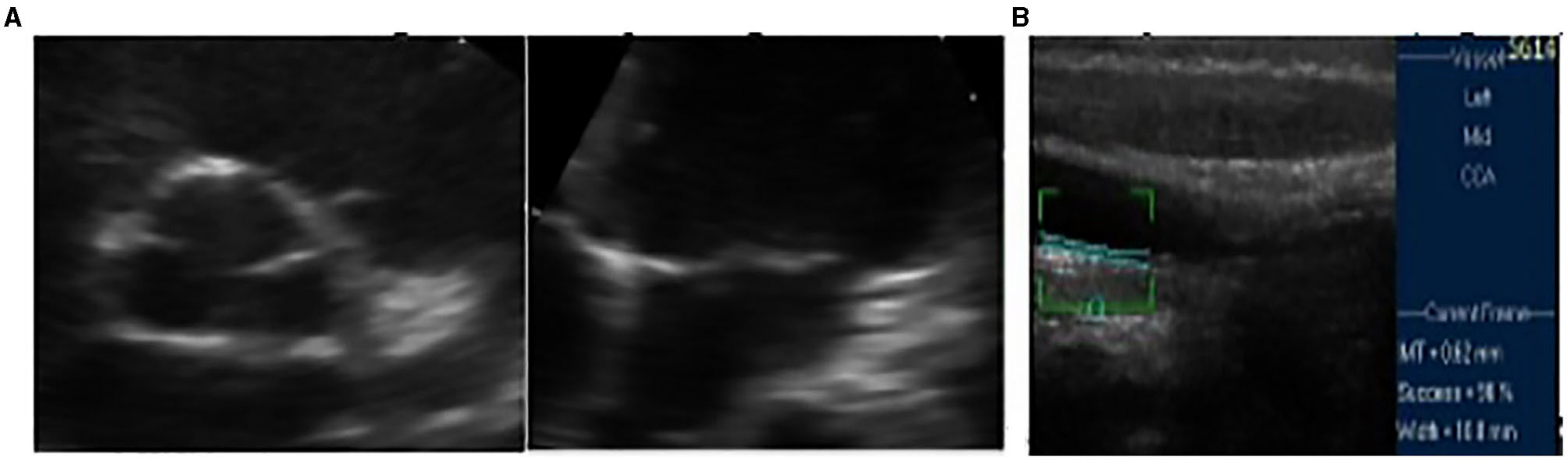
**(A)** Initial fibrocalcinous changes on the aortic root and on the mitral valve annulus. **(B)** Initial intima media thickness of the last 10 mm of the right common carotid artery.

Functional testing, electrocardiography at rest, and cardiopulmonary testing registered no abnormalities. Echocardiography revealed posterior annular calcification of the mitral valve and thickened and sclerotic aortic valve without any pathological gradient, normal ejection fraction (67%), and normal global longitudinal strain (−25%) (Figure 2). Additional tests for subclinical atherosclerosis were carried out in accordance with the European guidelines (7). Doppler ultrasound of the arteries of the extremities was performed by an angiologist and revealed no atherosclerotic lesions. Intima-media thickness of the carotid arteries was elevated for the patient's age and corresponded to the values of a 30-year-old male (Figure 2).

### Treatment Approach

A multidisciplinary approach was applied with the help of a pediatrician, cardiologist, and nutritionist to reduce the total cardiovascular risk. It also included dietary recommendations and motivation for regular physical activity and psychological support to comply with the long-term treatment (4, 5).

**Figure 3:**
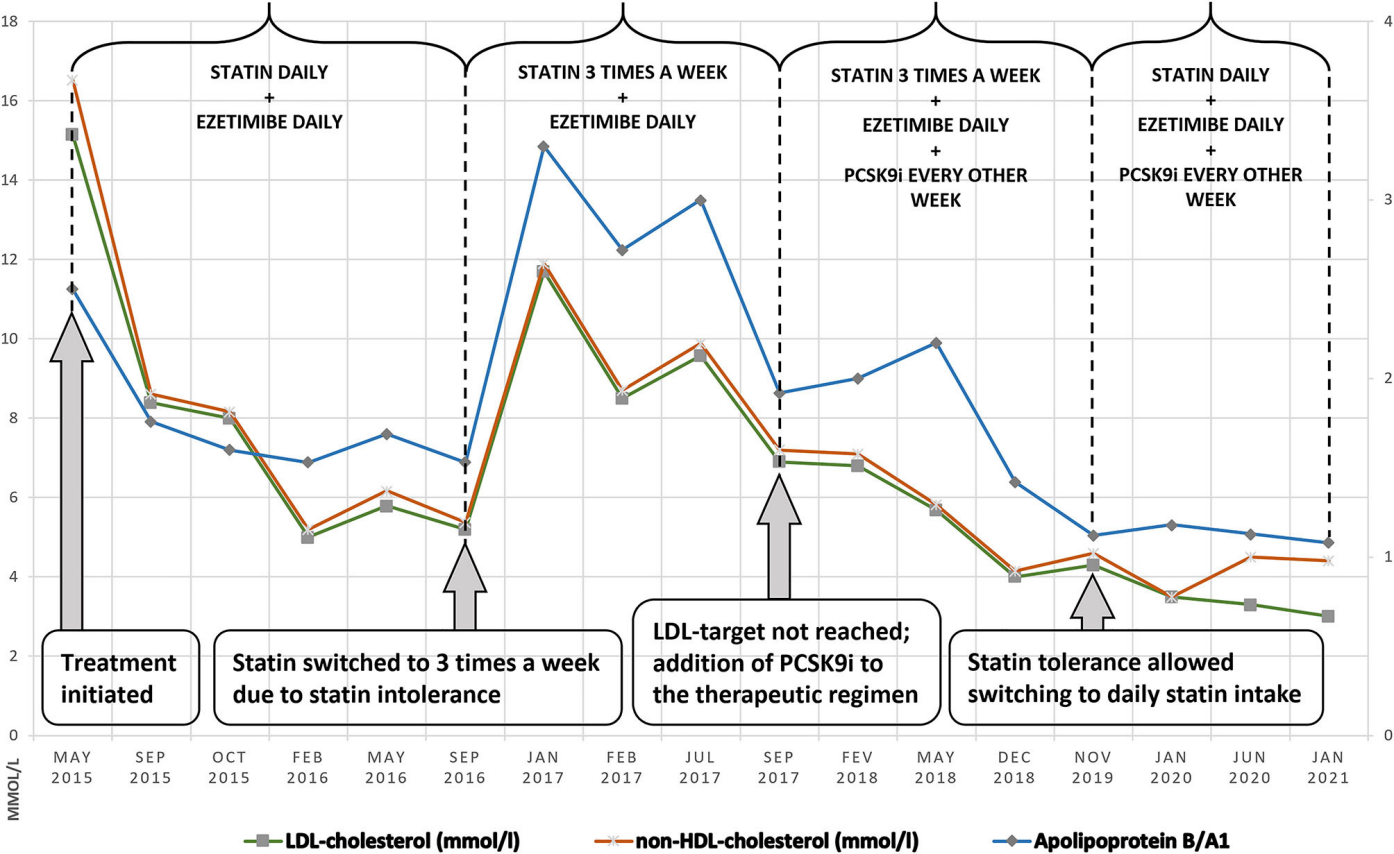
Dynamics of LDL-C, non-HDL-C, Apolipoprotein B/A1 during the 5-year follow-up.

A treatment plan for the patient was prepared in accordance with the European recommendations: administration of statins in the maximum tolerated dose and a cholesterol absorption inhibitor (3, 7) Initial treatment with Atorvastatin 20 mg in combination with Ezetimibe 10 mg was selected (8). With its help, the patient's LDL level decreased by roughly 46% for 5 months; however, he developed a severe statin intolerance: muscle weakness in his lower limbs and unwillingness to exercise. Creatin phosphokinase was elevated—approximately three times higher than the upper reference value—an insignificant finding in this case. A significant increase of the hepatic enzyme (alanine aminotransferase) levels was observed: a nearly threefold rise in comparison to baseline levels. Apart from the BMI, no other cause, potentially accountable for the statin intolerance (such as hypothyroidism, low vitamin D_3_, or impaired kidney function) was identified. Atorvastatin was replaced with Rosuvastatin 10 mg, but the symptoms persisted. During the following year, the patient was on a treatment with Ezetimibe 10 mg only and refused to take statins, which led to an increase in the LDL-C (Figure 3).

In cases of HoFH, LDL apheresis is advisable. Unfortunately, the procedure is not performed in Bulgaria due to its high cost (9). Treatment with Mipomersen or Lomitapide was considered, but it was turned down due to its high financial cost (10).

We adapted an intermittent regimen for the administration of Rosuvastatin: 10 mg three times a week with a daily intake of Ezetimibe 10 mg. As a result, myalgia disappeared. The follow-up showed varying levels of LDL-C, which were far from the desired target of 5.2–11.7 mmol/l (Figure 3). According to ESC/EAS Guidelines for the Management of Dyslipidemias, genetic testing of children is required for accurate determination of the necessary level of aggressive lipid-lowering therapy (3, 4). In May 2017, a significant development of the case was brought in by genetic analysis: next-generation sequencing and CNV analysis of a dyslipidemia panel of 29 genes (with sensitivity of more than 99%) were performed at the Department of Clinical Genetics, Academic Medical Center at the University of Amsterdam, Netherlands. Two pathological mutations within the *LDLR* gene were identified: c.1519A>G; p.(Lys507Glu) and c.2403_2406del; p.(Leu802Alafs^*^126). These mutations had been previously identified only in France and the Netherlands in a small number of cases: 13 for the first mutation and three for the second one (11). No additional pathogenic mutations were identified in the remaining 28 genes of the dyslipidemia panel. The analysis confirmed the initial suspicion of HoFH. The condition was in its more common form: compound heterozygosity. The clinical severity of compound heterozygosity is considered almost equal to that of true homozygosity (11). Molecular genetics confirmation strengthened our patient's determination to be more willing to accept new treatment strategies.

In September 2017, the boy, already 12 years old, became eligible for treatment with PCSK9i (12–14). His case was reviewed by a National Committee, consisting of a cardiologist, a pediatrician, and an endocrinologist, and he was approved for adjuvant biweekly treatment with Evalocomab 140 mg with the consent of his parent. The first follow-up result was obtained at the beginning of February 2018 at week 4 after two administrations of the drug. No reduction in the lipid levels was found: Total cholestrol was 8.8 mmol/l, and LDL-C was 6.8 mmol/l. Follow-up results at the beginning of May 2018 (nearly 6 months after the onset of the PCSK9i therapy) showed a reduction: total cholesterol was 7.46 mmol/l (15.2% reduction), and LDL-C was 5.68 mmol/l (16.4% reduction). Treatment with PCSK9i was combined with a maximum tolerated dose of Rosuvastatin 10 mg three times a week and Ezetimibe 10 mg daily. He was able to tolerate statins daily (BMI = 24.5 kg/m^2^) due to the sufficient values of growth variables (height, weight, and BMI) characteristic for the patient's age (12). Subsequently, the patient started taking a combined pill of 10 mg Rosuvastatin and 10 mg Ezetimibe daily along with Evalocomab 140 mg twice a week. Follow-up evaluation was performed every 6 months. LDL-C follow-up showed a tendency for reaching the target levels. A positive result of LDL-C decrease by 50% in comparison with the initial values was achieved after the addition of Evalocomab to the dual lipid-lowering therapy. This was the reason why an increase in the statin dose was not necessary. Since January 2020, the target levels have been successfully preserved below 3.5 mmol/l. (14) (Figure 3).

The dynamics of the different lipid profile values during the 5-year course are displayed in Figure 3. The mean reduction of LDL-C by a therapeutic approach that included Rosuvastatin 20 mg daily, Ezetimibe 10 mg daily, and Evolocumab 140 mg every 2 weeks led to an initial decrease in LDL-C by 41.17% during the first year. The effect increased over time, reaching 52.17% for the whole 2-year period. The effect on LDL-C for this period increased, which indirectly indicated lack of anti-Evolocumab antibodies (the latter have not been tested). No effect on triglycerides and HDL-C was observed. As a result of the triple lipid-lowering therapy, significant reduction in the levels of Apolipoprotein-B was detected, reaching 29.16%. No statistically significant difference was found in Lp(a) levels (*p* > 0.05).

Additional laboratory assays, such as complete blood biochemistry (blood glucose and HbA_1_c) and hematology analyses were carried out. The only ascertained increase was that of aminotransferase levels (three times the normal value) during the initiation of the daily statin intake. Subsequently, aminotransferase levels returned to normal.

**Figure 4:**
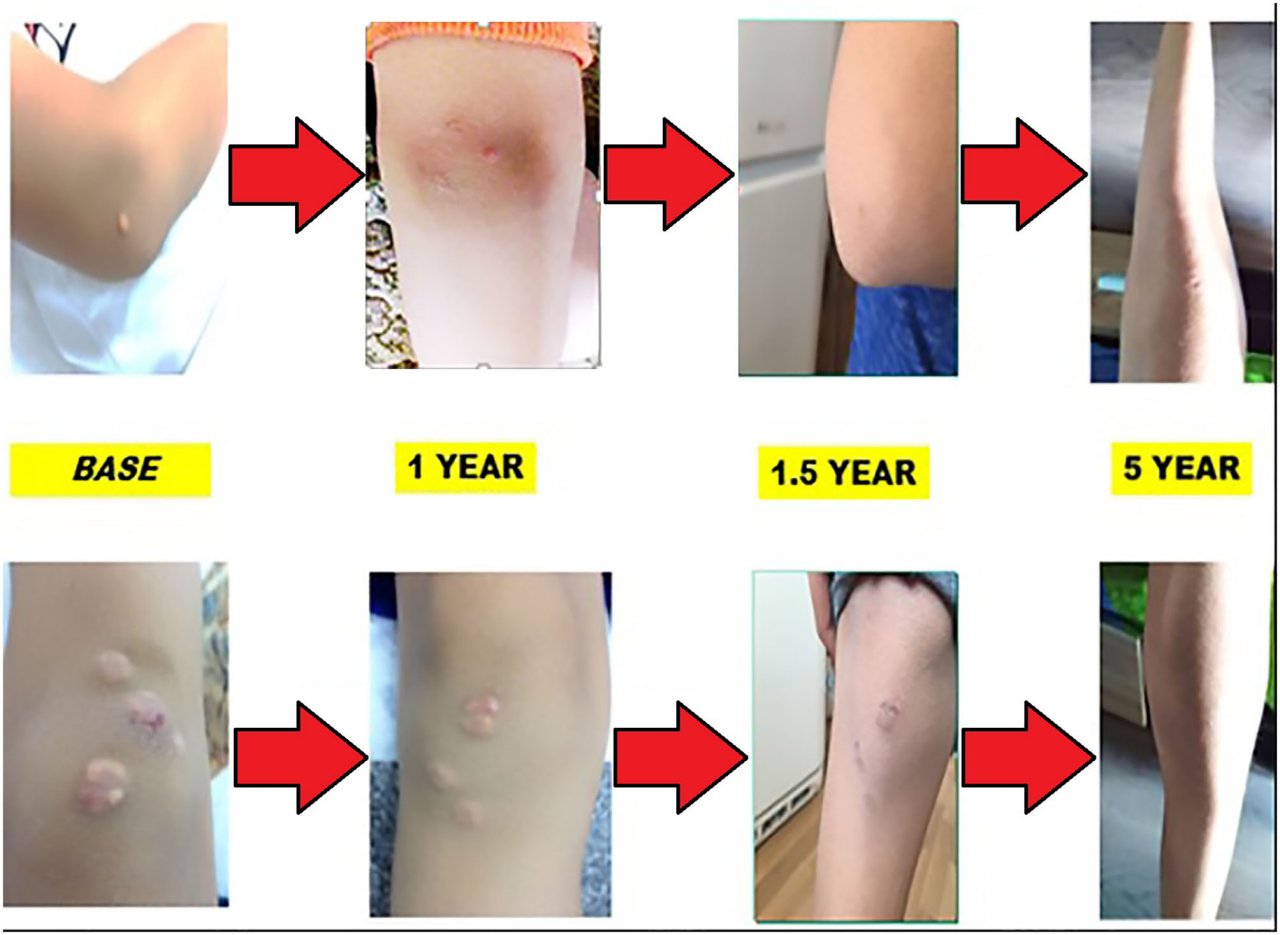
Involution of the xanthomas during the 5-year follow-up.

Each year, the patient undergoes a complete cardiological examination: electrocardiography, echocardiography, duplex sonography of the carotid arteries, and stress test. No abnormalities were detected, apart from the thickness of the communal carotid artery. During the treatment, the thickness showed no alteration. Involution of xanthomas was observed in the first year of treatment with a reduction of LDL levels by almost 50%. Skin lesions' appearance during the 5-year period is shown on Figure 4.

Side effects, including minor flu-like symptoms, were reported by the patient's mother after the first administration of Evolocumab. An increase in the incidence of upper respiratory tract infections has been observed since the addition of the monoclonal antibody to the therapeutic scheme.

During the ongoing treatment and monitoring, it was ascertained that the child had a normal growth rate and height, which was at the 50th percentile. BMI showed the following deviations: an initial increase in BMI, which was insignificant, followed by a reduction of body mass and reaching a BMI at the third percentile at the age of 15, which requires attention and subsequent follow-up (Supplementary Figure 1). Because of this fact, we planned an increase in energy intake and scheduled a consultation with a pediatric dietologist. Pubertal development was at Tanner Stage 1 at the beginning of the treatment. At the age of 15, the patient was at Tanner stage 3, which was interpreted as normal development. In January 2021, while being on a triple lipid-lowering therapy, the patient suffered a mild COVID-19 infection, which had no complications.

## Discussion

Treatment of HoFH is often difficult despite the advances in lipid-lowering therapies (3–5). The 2016 ESC/EAS Guidelines for the Management of Dyslipidemias suggest that a healthy lifestyle and diet should be adopted by children suffering from FH, and statin treatment should be initiated as early as 8 years of age. The control of LDL-C levels is considered to be crucial. Recommended goals for children >10 years of age are LDL-C <3.5 mmol/l (135 mg/dl) or at least a 50% reduction (3–5).

Potent statins, even at high doses, have proven to be insufficient for reaching the LDL-C targets with a reduction by 10–25% only detected in the majority of patients with HoFH. Nonetheless, they are in the basis of FH treatment (3). The addition of the cholesterol absorption inhibitor Ezetimibe lowers LDL-C further by 10–15%, adding to an overall reduction of 30–40% (3). Lipoprotein apheresis is a well-established treatment of both true homozygous (patients with two identical mutations, each in one of the copies of a certain gene) and compound heterozygous (patients with two different mutations, each in one of the copies of a certain gene) HoFH forms as well as of double heterozygous (patients with two different genes having a mutation in one of their copies) forms of FH. The high price and lifestyle requirements limit the administration of this treatment (9). Attention is currently directed at new available lipid-lowering therapies, including Lomitapide, Mipomersen, and PCSK9i (10, 12). Both PCSK9i, Evalocomab and Alirocumab, have proven to reduce LDL-C additionally by up to 60% when added to a standard lipid-lowering therapy in heterozygous FH patients with relatively minor side effects (12–14). In HoFH (both true homozygous and compound heterozygous) and double heterozygous FH patients, the reduction at week 12 is reported to be 30.9% for Evalocomab and 41.2% for Alirocumab (15). The literature review reveals a limited use of PCSK9i in children. In the TESLA Part B trial, which included 49 individuals with HoFH over the age of 12, Evalocomab was administered in eight children, the youngest of whom was 13 years old. Two of the children were true homozygotes, and six were compound heterozygotes for *LDLR* mutations. Therapeutic reduction in cholesterol levels depended on the severity of the identified mutations and the residual activity of the *LDLR* (16). There are many other potential causes for interindividual differences in patient responses. Observations from the reported case detect a double LDL-C reduction after the addition of PCSK9i to the therapeutic scheme: during the first 6 months, reduction was by 28%; at the end of the second year, the reduction was by 52%. This demonstrates the applicability of PCSK9i in young patients with FH. The further decrease in LDL-C levels after the addition of PCSK9i to the treatment regimen suggests that there is a residual activity of the LDL receptor in our patient. Considering the results from the genetic analysis (two different *LDLR* mutations, both rare, and hence hard to reliably correlate to a specific *LDLR* activity phenotype), we assume this is indeed true; probably at least one of the mutations is “receptor-defective” and encodes a product with a partially preserved activity (as opposed to the “receptor-negative” mutations that cause complete loss of function of the product).

Our observations also confirm the TESLA Part B trial data on *LDLR* compound heterozygous HoFH of 23 patients, in which LDL-C reduction (Evolocumab compared with placebo) ranges from a mean 40.8% in 57% (28/49) of patients with either one or two mutations in *LDLR* to no response in patients with two negative or suspected negative mutations. It should be mentioned that, in the TESLA Part B trial, patients were monitored for a significantly shorter period: only 24 weeks compared with the 2-year follow-up of our case. Based on both sources, we can assume that response to Evolocumab was related to the specific underlying genetic defect causing HoFH. Furthermore, we presume that the therapeutic effect becomes more pronounced with time as observed during the 2-year PCSK9i treatment period in our patient.

In our case report, PCSK9i treatment is the main cause for reaching LDL-target levels in combination with a statin and ezetimibe. The reason behind this could be partially attributed to one or both of the following: lack of genetic defect in the *PCSK9* gene and/or residual LDLR activity. The LDL-C reduction by 52.17%, owing to the addition of PCSK9i to the therapeutic regimen, would not be sufficient by itself to reach the LDL target in HoFH when LDL-C should be even lower. The use of a triple lipid-lowering therapy employs several different mechanisms for the lowering of LDL-C, which have a more potent effect when combined and achieve significantly lower LDL-C values.

The current case study supports the results from TESLA part 2 by reporting that genetic information provides incremental insight into homozygous and compound heterozygous LDLR mutation–caused HoFH and its possible response to treatment. We also agree that the response to Evolocumab depends on the specific genotype causing HoFH with a greater reduction in LDL-C recorded in patients with *LDLR* receptor-defective mutations compared with patients with *LDLR* receptor-negative mutations (12). Therefore, it can be concluded that, in a clinical trial setting, the genetic confirmation of HoFH is a valuable source of information for assessment of the genotype–phenotype correlations. Genetic results could also prove useful in the selection of pediatric patients eligible for Evolocumab treatment in clinical practice.

This clinical case demonstrates that the triple lipid-lowering combination in a patient with compound heterozygous HoFH is a good therapeutic option for reaching the target levels as a result of reduction in LDL-C levels by more than 50% in a pediatric patient. The compound heterozygosity in our reported FH case appears not to influence the efficacy of Evolocumab with LDL-C reductions in the same range as reported for the overall pooled analysis of FH patients from an Evolocumab phase 3 trial. The LDL-C-lowering activity of Evolocumab in FH, caused by compound heterozygous LDLR genotypes, is likely to be attributable to the presence of at least one partially functional allele (receptor-defective mutation) (15).

Lp(a) is a marker that predicts and stratifies the risk of atherosclerotic cardiovascular disease in adults with FH in combination with the LDL-C level, but its role in children is debated (17, 18). Its high initial levels in our case report are consistent with previous studies suggesting that high Lp(a) levels in children may be associated with a family history of atherosclerotic cardiovascular disease (18). Lp(a) testing may also identify children with FH who could benefit from a more aggressive management to reduce atherosclerotic cardiovascular disease risk. The lack of Lp(a) reduction in our patient is discordant with the results of previous studies reporting that, in patients with either one or two defective *LDLR* alleles, Lp(a) reduction was statistically significant (16). Currently, data in regard to Lp(a) being better than LDL in stratifying the cardiovascular risk in children is lacking (18).

The lack of decrease in Lp(a) after the addition of Evolocumab is still a disappointing and disconcerting result. Data on Lp(a) in the pediatric population is limited. Previous studies show a decrease in Lp(a) but in adults and as a secondary prevention (19, 20). It should be kept in mind that the prognostic benefits that PCSK9i has in relation to Lp(a) are mainly derived from secondary analysis and not as a main goal of studies. On the other hand, we still do not have enough data on the effects of PCSK9i in primary prevention in both adult patients and children with elevated Lp(a) levels. We can suggest that the lack of effect detected in our study supports the data in the literature that Lp(a) clearance is mediated by multiple receptors (21). Most likely due to the latter, even effective LDL-C-lowering pharmacotherapies, specifically enhancing the activity of hepatic LDL receptors, are not sufficient for an effective lowering of Lp(a) levels. This possible explanation would prove the lack of association of Lp(a) levels with the activity of any single one of the receptors expected to mediate hepatic removal of Lp(a). Targeting receptor-mediated removal of circulating Lp(a) particles for therapy may indeed require a complex approach. In accordance with the latter, recent efforts have been aimed at reducing the hepatic production rate of apolipoprotein (a). One realistic therapeutic strategy for our patient is the future use of the already approved RNAi-based PCSK9 inhibitor Inclisiran. The data from the literature proves the dose-dependent Inclisiran-mediated lowering of LDL-C and Lp(a) (22). Our team is hopeful that novel lipid-lowering therapies will help us reach the target levels of both LDL-C and Lp(a) and reduce the cardiovascular burden in the reported young individual.

Another significant problem is the thrombogenic risk associated with the persistent high levels of Lp(a) (23). It calls for a discussion on the need for antiplatelet prophylactic therapy (24). After examination of the other coagulation factors—platelets, fibrinogen, D-dimers—which were within the reference ranges and taking into account the normal vascular status, we decided to refrain from an antiplatelet treatment at this stage. We continue to monitor this issue.

Our patient had a mild COVID-19 infection a few months ago. That could be attributed to a sufficient immune response (even with Evolocumab). A few reasons can be given for the mild clinical course of the COVID-19 infection in our patient. First, statin therapy, due to its numerous pleiotropic effects on the vessel wall, may play a role because COVID-19 is an endothelial disease (25). Second, the initially elevated HDL-C levels, which remained high during the infection (HDL-C levels: 1.4 ± 0.15 mmol/l from the beginning and throughout the entire 5-year observation) probably have a connection with the administered lipid-lowering therapy: statin and PCSK9i (26, 27). Literature data suggest that PCSK9i treatment results in a more atheroprotective HDL particle profile; it decreases plasma levels of extra-large HDL particles and increases plasma levels of medium-sized HDL particles (27). The latter ones, when in high levels, are reported to be associated with a milder COVID-19 infection course (28). Similar data can be found in another observational study in which individuals with lower levels of HDL-C before the pandemic are at higher risk of suffering a severe COVID-19 infection (29, 30).

## Conclusion

The response to Evolocumab was related to the underlying genetic defect causing HoFH with the compound heterozygous LDLR genotype. We confirm the observations of previous studies that, in compound heterozygous HoFH patients who receive stable background lipid-lowering treatment and do not undergo apheresis, biweekly treatment with Evalocomab 140 mg was well tolerated and significantly reduced LDL-C. More information is needed for the use of PCSK9i in children as far as effectiveness and possible short- and long-term side effects are concerned. The new upcoming therapies will most likely help resolve the problem of the persisting high Lp(a) levels in our patient.

## Learning Objectives

- With the current limited literature data on the usage of PCSK9i in children with FH, the study contributes by providing additional information on the efficiency and safety within a 2-year period.
- On the other hand, the response to Evolocumab was related to the underlying genetic defect causing HoFH with compound heterozygous *LDLR* genotype. Compound heterozygous HoFH patients who receive stable background lipid-lowering treatment and do not undergo apheresis, biweekly treatment with Evalocomab 140 mg was well-tolerated and significantly reduced LDL-C for the reported period of 2 years.
- The persistent high Lp(a) level, even with the use of PSCK9 inhibitors in HoFH, is still a therapeutic challenge that we hope the new upcoming therapies will help us resolve.
- High HDL-C levels could be one of the reasons for the mild clinical COVID-19 infection course, a suggestion supported by recent literature data.

## Reporting Guidelines

The case report follows the consensus-based clinical case reporting guideline (CARE) checklist (30).

## Data Availability Statement

The datasets presented in this study can be found in online repositories. The names of the repository/repositories and accession number(s) can be found in the article/Supplementary Material.

## Ethics Statement

The only living parent of the child signed an informed consent to participate in the study, genetic analysis, and data publication.

## Author Contributions

LV-K expert in the field of FH, aided in the diagnostic and therapeutic process, led the writing of the publication. SK constructed the family tree, provided technical support in the photo development and in writing the abstract. MG provided additional interpretation of genetic analyses data and was responsible for the proofreading from medical genetics' standpoint. LC-B a pediatric cardiologist conducted the diagnostic tests, built the growth charts and monitored the pubescent development and treatment of the child. All authors have read and agreed to the published version of the manuscript.

## Conflict of Interest

The authors declare that the research was conducted in the absence of any commercial or financial relationships that could be construed as a potential conflict of interest.

## Publisher's Note

All claims expressed in this article are solely those of the authors and do not necessarily represent those of their affiliated organizations, or those of the publisher, the editors and the reviewers. Any product that may be evaluated in this article, or claim that may be made by its manufacturer, is not guaranteed or endorsed by the publisher.

## Supplementary Material

Supplementary Figure 1 BMI dynamics during the 5-year follow-up.
