# Supplementary figures and images for: Case Report: Difficulties in the Treatment of a 12-Year-Old Patient With Homozygous Familial Hypercholesterolemia, Compound Heterozygous Form − 5 Years Follow-Up
Source: Front Cardiovasc Med. 2021 Oct 8;8:743341. doi: 10.3389/fcvm.2021.743341 (PMC8531482; doi:10.3389/fcvm.2021.743341)

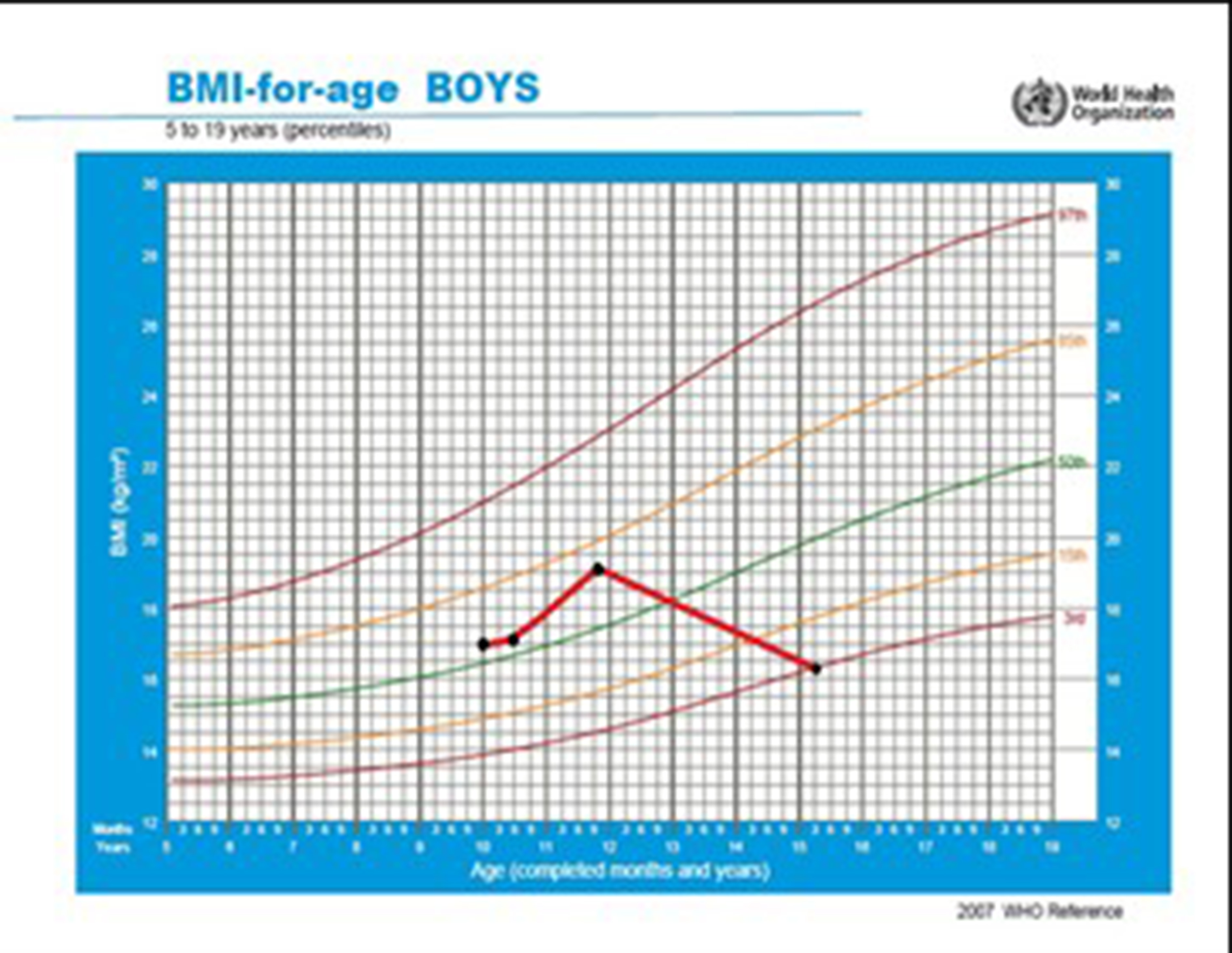

Supplement: Supplementary Figure 1 — BMI dynamics during the 5-year follow-up. [file Image_1.JPEG]
